# Facile and Gram-scale Synthesis of Metal-free Catalysts: Toward Realistic Applications for Fuel Cells

**DOI:** 10.1038/srep08376

**Published:** 2015-03-02

**Authors:** Ok-Hee Kim, Yong-Hun Cho, Dong Young Chung, Min Jeong Kim, Ji Mun Yoo, Ji Eun Park, Heeman Choe, Yung-Eun Sung

**Affiliations:** 1 Department of Science, Republic of Korea Naval Academy, Jinhae-gu, Changwon 646-797, South Korea; 2 Department of Chemical Engineering, Kangwon National University, Samcheok 245-711, South Korea; 3 Center for Nanoparticle Research, Institute for Basic Science (IBS), Seoul 151-742, South Korea; 4 School of Chemical and Biological Engineering, Seoul National University, Seoul 151-742, South Korea; 5 School of Advanced Materials Engineering, Kookmin University, Seoul 136-702, South Korea

## Abstract

Although numerous reports on nonprecious metal catalysts for replacing expensive Pt-based catalysts have been published, few of these studies have demonstrated their practical application in fuel cells. In this work, we report graphitic carbon nitride and carbon nanofiber hybrid materials synthesized by a facile and gram-scale method via liquid-based reactions, without the use of toxic materials or a high pressure-high temperature reactor, for use as fuel cell cathodes. The resulting materials exhibited remarkable methanol tolerance, selectivity, and stability even without a metal dopant. Furthermore, these completely metal-free catalysts exhibited outstanding performance as cathode materials in an actual fuel cell device: a membrane electrode assembly with both acidic and alkaline polymer electrolytes. The fabrication method and remarkable performance of the single cell produced in this study represent progressive steps toward the realistic application of metal-free cathode electrocatalysts in fuel cells.

Although the first fuel cells were fabricated in 1839 by William Grove, fuel cell-based technology has still not become fully commercialized. One of the main impediments to the commercialization of fuel cells is the use of expensive Pt catalysts. Significant effort has been devoted to replacing the expensive Pt-group metal (PGM)-based catalysts used in the oxygen reduction reaction (ORR) with inexpensive, more abundant nonprecious metal catalysts^1^. The progressive steps that have been taken in the development of ORR electrochemical catalysts can be summarized as follows: (1) Reduction of the size of Pt catalysts to nm scale, with a concomitant increase in their surface area and efficiency; (2) fabrication of Pt-based alloys or core-shell structures for enhanced activity and stability; (3) replacement of Pt-based catalysts with cheaper and non-PGM compounds such as those based on Fe or Co; and (4) implementation of metal-free materials such as N-doped carbon^2^,^3^.

Currently, the most promising candidates are transition metal–nitrogen materials, despite the drawbacks associated with their cost, activity, and stability. Moreover, controversy has surrounded the role of metals in ORR catalysts^4^,^5^,^6^,^7^. Alternatively, the intrinsic catalytic properties of nonmetallic N-doped carbon materials, such as N-carbon nanotubes (N-CNTs), N-graphene, and graphitic carbon nitride (g-C_3_N_4_), have also attracted interest. However, the ORR mechanisms and associated active sites of such materials (e.g., pyridinic N, pyrrolic N, and graphitic N) are still under debate^8^,^9^,^10^.

Recently, g-C_3_N_4_ has proven to be effective as a multifunctional catalyst in various applications^11^,^12^,^13^,^14^. In particular, its ORR catalytic activity is considered to be significant for clean energy conversion and storage applications. g-C_3_N_4_ has numerous advantages compared with traditional Pt catalysts, including (1) relatively lower costs and greater abundance, (2) increased stability toward CO poisoning, (3) greater methanol tolerance, and (4) the possibility of obtaining a variety of nanostructures via a templating method. In addition, g-C_3_N_4_ has a higher nitrogen content and more active reaction sites compared to other N-carbon materials, resulting in better performance as a practical metal-free ORR electrocatalyst^8^,^11^,^12^,^13^,^14^. Various methods have been developed for the synthesis of g-C_3_N_4_. A solid-state reaction at high pressure and temperature and a poly-condensation reaction of liquid precursors such as cyanamide are the traditional methods used to synthesize bulk g-C_3_N_4_. However, the bulk-phase reaction has been demonstrated to be prone to incomplete condensation of the precursors^11^, and cyanamide is not only expensive but also highly explosive and toxic^14^. Consequently, although some of g-C_3_N_4_-type catalysts are still being proposed, their use is not practical.

Herein, we report the facile and gram-scale production of a g-C_3_N_4_ hybrid material (denoted hereafter as g-CN) via a liquid-based reaction without the use of cyanamide or a high-pressure reactor. The resulting composite material prepared using a metal-free procedure exhibited a fuel cell cathode catalytic activity competitive with that of a commercial Pt/C catalyst. Furthermore, as illustrated in Fig. 1, g-CN exhibited outstanding performance in membrane electrode assemblies (MEAs) of polymer electrolyte membrane fuel cells (PEMFCs, in which protons are the conducting species) and anion exchange membrane fuel cells (AEMFCs, in which hydroxide ions are the conducting species).

## Results

### Fabrication of g-CN and g-CN-CNFs

**Figure 2:**
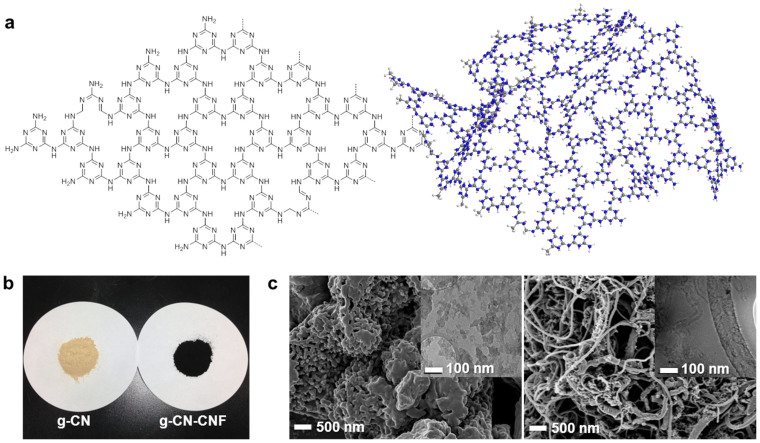
Structure and image of g-CN and g-CN-CNF. (a) Chemical structure and schematic diagram of g-CN; (b) photograph of g-CN and g-CN-CNF; and (c) FE-SEM images of g-CN (left) and g-CN-CNF (right) (insets: TEM images).

A schematic illustration of the g-CN and photographs of the bulk g-CN and g-CN with carbon nanofibers (CNFs) are presented in Figs. 2a and 2b, respectively (see Fig. S1 in the Supplementary Information for experimental details). The g-CN was synthesized from melamine and cyanuric chloride, which are nitrogen-rich materials. Melamine is a commonly used in industrial applications such as fireproofing materials and melamine resin. Additionally, melamine was recently used as a nitrogen precursor for the preparation of N-doped carbon materials^15^,^16^,^17^. Cyanuric chloride is an important starting material for the synthesis of dendrimers^18^,^19^, in which an iterative substitution reaction of cyanuric chloride and an amine group is induced via the sequential addition of amines and under controlled temperature. Tetrahydrofuran is commonly used as a solvent for the substitution reaction of cyanuric chloride. However, *N*,*N*-dimethylmethanamide was used as a solvent in this study because of its high boiling point.

During the stepwise synthesis, melamine and cyanuric chloride produced the g-CN structure. Such a condensation reaction is possible because of the different reactivities of trichlorotriazines and cyanuric chloride. At low temperature (i.e., 0°C), only one site of triazine can be substituted, whereas two sites can participate in substitution reactions that occur at room temperature (25°C), and all three sites can react under elevated temperatures (greater than 70°C). *N*,*N*-Diisopropylethylamine (Hünig's base) was used as a non-nucleophilic base for the substitution reaction because its molecular structure makes it ideal for this application. Specifically, the proton attached to the nitrogen atom is shielded by steric hindrance, which makes Hünig's base a good base but a poor nucleophile. In the last condensation step, CNF was added as a conducting carbon material to increase the electrical conductivity. CNF as a support material can easily form secondary and these interconnected pores indeed exist between the catalyst agglomerates, ranging in size from ~200 nm to 1 μm (Figs. S2, S3). Therefore, CNF can facilitate mass transfer within the thick catalyst layer. The chemical reaction in the electrode of a PEMFC has been reported to occur primarily in the secondary pores rather than in the primary pores^20^. Prior to use, CNF was treated with acid (H_2_SO_4_:HNO_3_ = 3:1 vol. %) for 7 h at 90°C to remove trace metal residues; the resulting sample is denoted as g-CN-CNF. A g-CN sample containing the intermediates was characterized by ^13^C and^15^N solid-state cross polarization-magic angle spinning (CP-MAS) NMR, X-ray diffraction (XRD), FT-IR spectroscopy, photoluminescence (PL) spectroscopy, and X-ray photoelectron spectroscopy (XPS).

**Figure 3:**
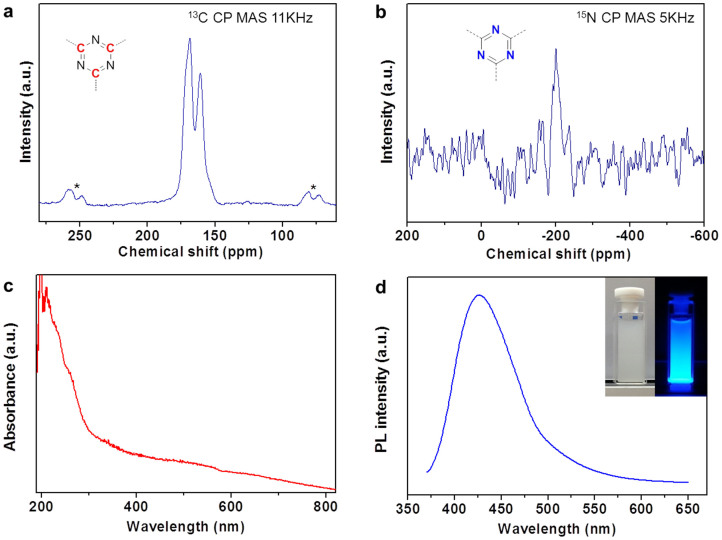
Physical characterization of g-CN. (a) ^13^C CP-MAS NMR spectrum of g-CN (spinning side-bands are denoted with asterisks); (b)^15^N CP-MAS NMR spectrum of g-CN; (c) absorption spectrum and (d) photoluminescence spectrum (inset) of g-CN under 350 nm excitation at 298 K. The insets are photographs g-CN in ethanol under natural light (left) and under UV irradiation at 365 nm (right).

The ^13^C and^15^N solid-state CP-MAS NMR spectra of g-CN are shown in Figs. 3a and 3b, respectively. Despite the fact that the g-CN in this study was not synthesized by a traditional method such as the condensation of precursors in the bulk phase under high pressure and high temperature used in the synthesis of poly(triazine), the NMR spectra were similar for the products synthesized using the two methods^18^,^19^. The ^13^C NMR spectrum of g-CN (Fig. 3a) shows two peaks at 161.0 ppm and 168.6 ppm. These chemical shifts are in agreement with those for sp^2^-hybridized carbon environments. In addition, the^15^N NMR data show a broad resonance signal between −185 ppm and −205 ppm, corresponding to the ring N atoms of g-CN. These values are typical for tertiary nitrogen atoms of triazine rings (Fig. 3b).

Furthermore, for the absorption and PL spectra (Fig. 3c and d), the conventional carbon nitride shows the typical absorption pattern of an organic semiconductor with a strongly expressed band gap adsorption at about 420 nm, and this is in good accord with the g-CN materials in absorption spectrum (Fig. 3c). And peaks centered around 420–430 nm were found in PL spectrum (Fig. 3d). The origin of this blue emission of g-CN under 350 nm excitation at 298 K attributed to the band-edge transition or the exciton combination. This is consistent with its pale yellow color, as previously reported^12^.

**Figure 4:**
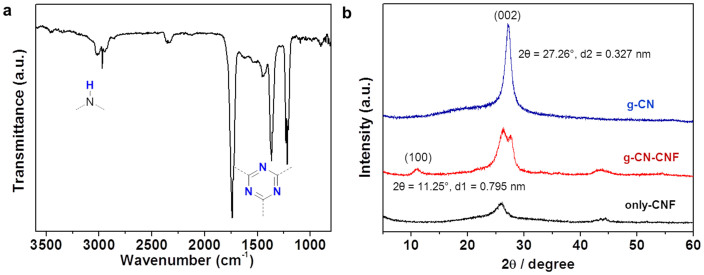
FT-IR spectrum and XRD pattern of g-CN materials. (a) FT-IR spectrum of g-CN, (b) XRD pattern of g-CN materials.

Additionally, in the FT-IR spectrum (Fig. 4a), the linkage of the triazine ring systems was confirmed by the appearance of major peaks in the 1200–1600 cm^−1^ region, which correspond to sp^2^C-N and sp^2^C = N. The absorption band at approximately 3100 cm^−1^ is attributed to the N-H stretching modes. In addition, the XRD pattern (Fig. 4b) and TGA trace (Fig. S5) of g-CN closely agree with those of other carbon nitride materials obtained by traditional synthesis methods^21^,^22^,^23^. XRD patterns of g-CN (Fig. 4b) representing the graphitic structure with an inter-planar stacking distance of 0.327 nm. The XRD pattern of the CNF and g-CN-CNF peak is added for comparison. The strongest peak of g-CN at 27.26° is a characteristic inter-planar stacking peak of aromatic systems, indexed for graphitic-like layer structure materials as the (002) peak. A slight broadening peak of the g-CN-CNF was detected at around 11.25°, corresponding to a distance of 0.795 nm and being indexed as (100), assigned to an interplanar distance between nitride pores. TGA weight loss curve of g-CN and g-CN-CNF measured in N_2_ with rate of 5°C/min (Fig. S5). During the pyrolysis step, almost all of g-CN thermally decomposed above 750°C. Pristine g-CN showed a 100% weight loss after 750°C while carbon showed no obvious weight loss, indicating no carbon contamination in carbon nitride. The TGA curves are still similar to that of a polymeric carbon nitride like g-C_3_N_4_.

### Electrochemical activity of g-CN and g-CN-CNFs

**Figure 5:**
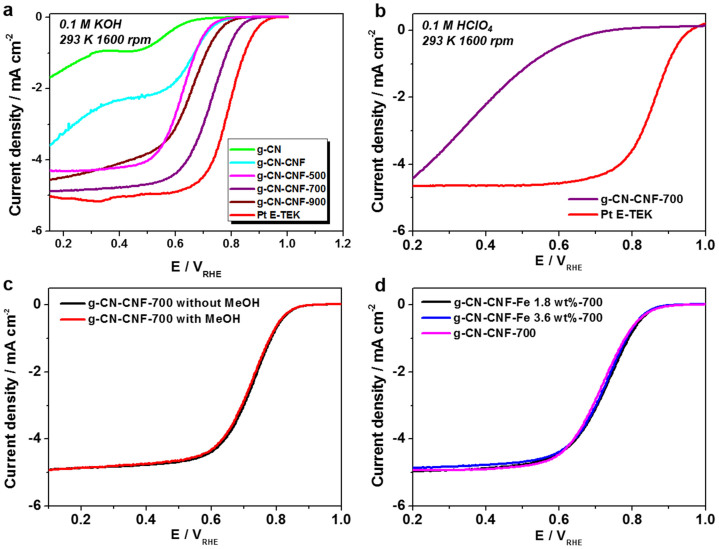
Electrochemical characterization of g-CN-CNF. (a) Linear sweep voltammograms (LSVs) on RDE at 1600 rpm under 293 K of a) g-CN-CNF pyrolyzed at various temperature in O_2_ saturated 0.1 M KOH solution, (b) g-CN-CNF-700 in O_2_ saturated 0.1 M HClO_4_ solution. (c) g-CN-CNF-700 with/without methanol, (d) g-CN-CNF-700 with/without metal.

The electrocatalytic activity of g-CN toward the ORR was evaluated by cyclic voltammetry (CV) (Fig. S5) and linear sweep voltammetry (LSV) (Fig. 5a). Compared with g-CN, g-CN-CNF, and the other samples with heat-treatment, the g-CN-CNF-700 catalyst revealed the most obvious ORR peak with a larger cathodic current, indicating a better electrocatalytic performance for ORR. Notably, the inherent ORR activity on pure g-CN is negligible. Therefore, a heat treatment was performed to increase this material's ORR activity. Although the literature generally agrees that pyrolysis beneficially affects both the activity and stability of non-Pt-based electrocatalysts, controversy remains over what aspect of the heat treatment is responsible for the positive effects^24^. Specifically, the enhanced ORR activity and stability is attributed to follow reasons: 1) catalyzing the formation of a special type of carbon, which is actually the active phase such as graphitic nitrogen and/or pyridinic nitrogen functional groups, 2) increasing the degree of edge plane exposure, 3) improving the dispersion of the supported material, 4) final carbon structure and moiety change such as increasing hydrophilicity and generation of micropores on the surface. The objective of this study, however, was not to investigate the precise mechanism of pyrolysis, but rather to develop high-performance catalyst materials using a facile synthesis method. Therefore, the ORR activity was measured without further probes into the role of pyrolysis.

The onset potential, which typically reflects the activity of the electrocatalyst, increased steadily as the temperature was increased to 700°C. However, the onset potential decreased at temperatures greater than 700°C because of a lack of active sites on g-CN, as indicated by the low nitrogen content of g-CN-CNF-900 (approximately 0.81 wt%) measured using an elemental analyzer (Table S1). Compared to g-CN, g-CN-CNF, and other samples treated by pyrolysis (denoted as g-CN-CNF-yyy, where yyy is the heat treatment temperature), the g-CN-CNF-700 catalyst exhibited the best electrocatalytic performance in 0.1 M KOH. The electron transfer number n of g-CN-CNF-700 was 3.46 according to the measured Koutecky–Levich plots (Fig. S6). Based on these plots, the electrocatalytic ORR mechanisms and dominated processes were further investigated. All plots show good linearity with various rotation speeds. Usually, the number of electrons transferred per O_2 _molecule (n) for ORR and kinetic current density can be obtained from the slope and intercept of Koutecky-Levich plots. The electron transfer numbers (n) of g-CN-CNF-700 were 3.46, 3.42, and 3.35 at 0.2, 0.3, and 0.4 V, respectively. This means that the ORR catalyzed by g-CN-CNF-700 was nearly dominated by the 4e^−^ pathway.

The electrocatalytic activity of g-CN-CNF-700 was also evaluated using a rotating disk electrode (RDE) under half-cell conditions in acidic solution (Fig. 5b), which indicated that its activity was very weak. However, the results obtained in an H_2_/O_2_ acid PEMFC test were fairly good, despite the poor behavior of the catalyst in acidic solution. This point will be discussed later.

Notably, in a single cell (the practical unit of fuel cells), the pH value is not constantly fixed at 1 or 14 as it is under half-cell conditions. Indeed, the neutral water supplied continuously from the fully humidified inlet gas forms/disappears at the catalyst interface during cell operation, especially during flooding. The pH at the catalyst interface, where the ORR reaction actually occurs, is less than 12 for alkaline fuel cells and greater than 1 for acidic fuel cells. Therefore, we assume that the activity of electrocatalysts in the half-cell is not always necessarily proportional to the performance of the single cell. The single-cell performance is discussed further in the next section.

An accelerated durability test (ADT) was conducted on the basis of the US Department of Energy (DOE) protocol (Fig. S7), and no activity loss was observed. Such excellent stability is an interesting feature of N-doped carbon-based catalysts^11^,^12^,^13^,^14^. Another important concern for cathode materials in PEMFCs is their tolerance toward methanol. A high catalytic selectivity for the studied cathode reactions against methanol oxidation is shown Fig. 5c. The ORR currents of g-CN-CNF-700 remained constant, irrespective of the presence of methanol. These data indicate that g-CN-CNF materials can serve as alternative electrocatalysts for cathodes in direct methanol fuel cells (DMFCs). The transition metal Fe was added into g-CN-CNFs before the last condensation step to evaluate the effect of metal dopants on the ORR activity. The amount of Fe precursor (FeCl_3_) was adjusted to 1.8 wt% and 3.6 wt% relative to g-CN-CNF. The metal content in non-PGM catalysts is well known to play an important role in catalyst activity and stability^2^,^24^. Contrary to this expectation, the results obtained for g-CN-CNF-700 with and without Fe indicate similar activities toward the ORR, regardless of the amount of Fe added (Fig. 5d). Several groups have reported that the transition metal itself does not function as an active site for ORR, but rather facilitates the incorporation of N-containing functionalities into graphitic carbon at high temperatures^11^,^25^,^26^. In contrast, in a g-CN structure, where the N-containing graphitic carbons already exist inside a carbon matrix prior to pyrolysis, the metal is not required. Therefore, we concluded that the addition of Fe dopants does not affect the electrocatalytic activity of g-CN-CNF. These results are comparable to those reported by other groups. For example, Silva *et al*. reported that the strongest activity was obtained when the catalyst contained no metal dopants^25^. However, a direct comparison of these results is not possible because of differences between the main objectives and experimental conditions of the two studies.

**Figure 6:**
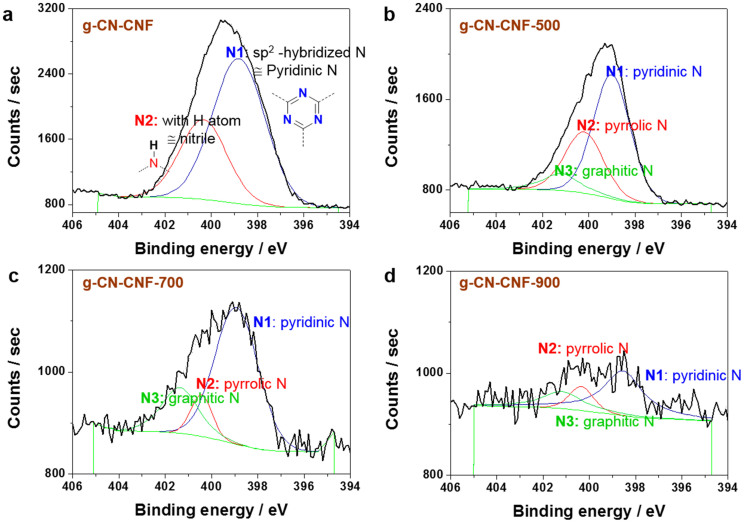
XPS spectra of N1s. (a) g-CN-CNF, (b) g-CN-CNF-500, (c) g-CN-CNF-700, and (d) g-CN-CNF-900.

The proposed basic explanation of the ORR mechanism is that the interaction of oxygen with the surface of the catalyst involves the d-band orbital energy of the metal atoms at the surface^12^,^27^. Because of the absence of d-bands in the atomic valence orbitals of metal-free catalysts, the tailoring of the electrode surface is thought to be critical. Toward this end, XPS was carried out (Fig. 6) to investigate the surface of the N-doped structure in greater detail. The N 1s peak positions are similar to those reported in previous studies^11^,^12^,^13^,^14^. The total N content and N composition ratio determined from the XPS spectra are summarized in Table S1. When combined with the data from the ORR activity (Fig. 5a) and XPS measurements, the results can be summarized as follows. First, the N content and ORR activity do not exhibit a linear relationship. The g-CN-CNF and g-CN-CNF-500 samples exhibited higher N contents compared to the other samples, although the best performance was exhibited by g-CN-CNF-700. Both Lai *et al*. and Biddinger *et al*. have also reported that the total nitrogen content does not play a critical role in determining the ORR activity^8^. Second, on the basis of the primary difference between the ORR polarization curves of the g-CN-CNF and g-CN-CNF-500/700/900 samples, graphitic N was inferred to facilitate a 4e^−^ pathway. The only g-CN-CNF with pure pyridinic N did not undergo pyrolysis and did not possess a graphitic N moiety. Therefore, the most reasonable interpretation is that pyridinic N only follows a 2e^−^ reduction mechanism for the ORR, consistent with the results of Luo's investigation on pure pyridinic N-doped graphene^10^.

Our findings are also similar to those reported by Silva *et al*., who reported that greater proportions of quaternary (graphitic) N centers favor a 4e^−^ process in the ORR^25^. Third, the g-CN-CNF-700 appeared to exhibit excellent electrocatalytic activity because it contained the highest proportion of pyridinic N. However, given the N proportion and pyridinic N content, g-CN-CNF-700 does not contain the largest amount of pyridinic N species. As a consequence, the question of whether the most efficient active sites are those of pyridinic N or graphitic N remains unanswered.

### Single-cell performance of a g-CN-CNF-based MEA

**Figure 7:**
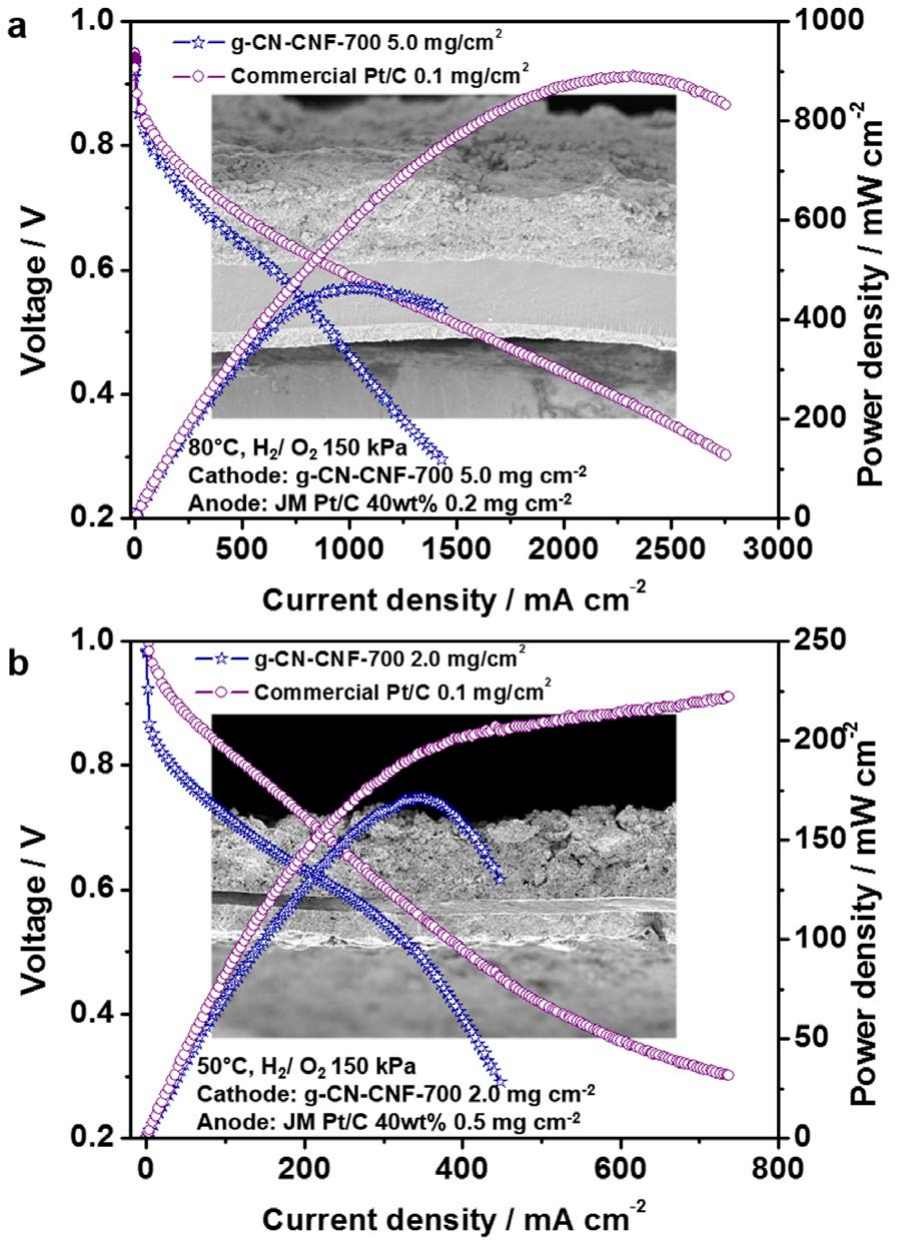
Polarization curves of g-CN-CNF-700-based MEAs. (a) Acidic fuel cell (PEMFC) with a cathode g-CN-CNF-700 catalyst loading of 5.0 mg cm^−2^ operated at 80°C; (b) alkaline fuel cell (AFC) with a g-CN-CNF-700 catalyst loading of 2.0 mg cm^−2^ operated at 50°C. The H_2_/O_2_ in the MEAs were fully humidified and supplied at a total outlet pressure of 150 kPa. Figures in the background are cross-sectional FE-SEM images of the g-CN-CNF-based MEAs.

The performance of the single cell using an MEA with g-CN-CNF-700 as the cathode catalyst is shown in Fig. 7. Both PEMFCs and AEMFCs were tested. In the case of the PEMFC, all of the polarization curves were obtained under DOE standard conditions (Fig. 7a). The current density at 0.6 V was 649 mA cm^−2^, which was 69% of that of an MEA fabricated using commercial Pt/C. More importantly, the maximum power density was 464 mW cm^−2^, which is the highest performance reported to date for a metal-free cathode catalyst in a practical device such as an MEA in a PEMFC. For example, Ding *et al*. reported a planar, N-rich, metal-free catalyst with a maximum power density of 320 mW cm^−2^.^6^ The g-CN-CNF-700-based MEA employed in this study exhibited better single-cell performance. To the best of our knowledge, such a high power density has not been previously reported for a metal-free catalyst. Given the rapid voltage drop in the high-current-density region, the g-CN-CNF-700-based MEAs appeared to suffer from gas transport loss because of the much thicker electrode (50–60 μm) used compared to that containing commercial Pt/C (5–10 μm) (Figs. S2 and S3). Therefore, further study was necessary to optimize the electrode structure to facilitate O_2_ transfer in the catalyst layer^28^,^29^. Additional analyses such as *in situ* electrochemical impedance spectroscopy (EIS) were conducted to evaluate the characteristics of the cell performance (Fig. S8). In general, the charge transfer resistance is primarily determined by the interfacial reaction kinetics, ionic conductivity, and diffusion limitations within the catalyst layer. The charge transfer resistance of g-CN-CNF-700 was larger than that of commercial Pt/C, and the former appeared to undergo severe mass transfer loss, probably because of its much thicker electrode.

Additionally, the performance of an AEMFC fabricated using the synthesized catalysts was also studied (Fig. 7b). The current density at 0.6 V was 248 mA cm^−2^, and the maximum power density was 171 mW cm^−2^. The performance characteristics of the g-CN-CNF-700-based PEMFC and AEMFC are summarized in Table 1. Whereas the performance of the AEMFC appeared to be inferior to that of the PEMFC, the current density of the AEMFC at 0.6 V was 80.5% of that of commercial Pt/C—greater than that of the PEMFC (69%). Some challenges remain for the AEMFC, all of which are related to the relatively low ionic conductivities, insufficient stabilities, fuel crossover, carbonation, and water management of the PEMs^30^. In addition, numerous problems regarding the optimization of the MEA fabrication process and fuel cell operating conditions still need to be resolved. If these challenges are overcome, a comparable result may be anticipated for PEMFCs.

## Discussion

We have demonstrated an approach for the facile and gram-scale production of g-CN materials and demonstrated the excellent performance of the g-CN-CNF-700-based MEA in a single cell of both a PEMFC and an AEMFC. The g-CN was synthesized by a liquid-based poly-condensation reaction, and a completely metal-free cathode material was obtained by pyrolysis. This newly synthesized material exhibited remarkable methanol tolerance, selectivity, and stability without the inclusion of a metal dopant. The graphitic N moiety appears to be responsible for the 4e^−^ process in the ORR. More importantly, the results of this study demonstrate for the first time that a completely metal-free fuel cell cathode can be used in a practical device, both in a PEMFC and an AEMFC, making it a potential candidate for the replacement of Pt-metal-based catalysts. Even better performance is expected to be achieved through optimization of the electrode structure to expedite mass transfer. The fabrication method and remarkable performance of the single cell in this study represent progressive steps toward realistic applications of metal-free materials in commercialized fuel cells.

## Methods

### Materials and Characterization

Commercially available solvents and reagents were used without further purification unless otherwise noted. The carbon nanofiber (CNF) was purchased from (Carbon Nanomaterial Technology Co., Gyongju, Republic of Korea). Both the ^13^C and the^15^N solid-state NMR spectra were recorded on Bruker Avance II instruments using a cross-polarization (CP) magic-angle-spinning (MAS) sequence mode. The samples were loaded into 4 mm ZrO_2_ rotors and spun at 11 kHz (5 kHz for ^15^N) with a pulse delay of 5 s and a contact time of 5 ms (3 ms for ^15^N). The data were referenced to trimethylsilane and ammonium chloride. In the spectra, resonances from the amino groups were barely observed because the NH signals were attenuated by the long polarization inversion time.^[8a]^ The morphology of the synthesized catalysts was characterized by high-resolution transmission electron microscopy (HR-TEM; JEOL 2010) and field-emission scanning electron microscopy (FE-SEM; Carl Zeiss, SUPRA 55 VP). The UV-visible and PL spectra were measured on a Scinco 2100 spectrophotometer and a Jasco FP-750 spectrofluorometer, respectively. X-ray diffraction (XRD) analyses of the prepared catalysts were carried out on an X-ray diffractometer (Rigaku D/MAX 2500) equipped with a Cu-Kα radiation source operated at 40 kV and 100 mA. The samples were scanned from 20° to 80° with a scanning rate of 2° min^−1^. X-ray photoelectron spectra (XPS) were obtained using an Al-Kα source (ESCALAB 250 XPS spectrometer, VG Scientifics). The binding energies were calibrated with respect to the C (1s) peak at 285 eV, and the experimental data were curve-fitted using the AVANTAGE 4.19 software package. The nitrogen content was determined using a CHN elemental analyzer (LECO Corp., US/CHNS-932). A thermogravimetric analyzer (TAC 7/DX) was used to analyze the thermal stability and evolved gases.

### Preparation of g-CN and g-CN-CNF

Melamine (1 eq.) was suspended in DMF and subsequently added to *N*,*N*-diisopropylethylamine. A solution of cyanuric chloride (1 eq.) in DMF was then added dropwise with stirring at 0°C in an ice-bath. After 1 h, the reaction temperature was increased to room temperature and the solution was stirred for an additional 5 h. The temperature was then decreased back to 0°C, and the solution of cyanuric chloride (1 eq.) in DMF was again added dropwise. After 1 h, the reaction temperature was increased to room temperature and the solution was stirred for an additional 5 h. Finally, the system was refluxed for 12 h under nitrogen, after which the flask was cooled to room temperature and the yellow-colored precipitate was collected by filtration. The product was washed with water and ethanol twice, and then dried under vacuum at 100°C. For g-CN-CNF, conducting materials such as CNF in DMF were added dropwise to the mixture prior to the reflux step. The mass ratio of the support carbon material CNF and g-CN was adjusted by approximately 70 wt%. For pyrolysis, g-CN-CNF was heated to the target temperature under Ar at a ramp rate of 100°C h^−1^ and was maintained at the target temperature for 30 min. For g-CN-CNF-Fe-700, iron (III) chloride (Sigma Aldrich, reagent grade, 97%) was added with stirring to a solution of g-CN intermediate solution before the last condensation step. The amount of the Fe precursor was adjusted by 1.8 and 3.6 wt% of g-CN and CNF. The mixture was stirred at room temperature for 2 h and CNF in DMF were added dropwise to the mixture prior to reflux. Finally the system was refluxed for 12 h under nitrogen. The flask was then cooled to room temperature, and the black-colored precipitate was filtered. The product was washed with excess water and ethanol three times and dried under vacuum at 100°C. For pyrolysis, g-CN-CNF-Fe was heated to the target temperature under Ar at a ramp rate of 100°C h^−1^ and kept at the 700°C for 30 min.

## Author Contributions

O.-H.K. conceived the idea and designed the experiments. O.-H.K., D.Y.C. and M.J.K. performed the experiments, analyzed the data, and wrote the manuscript with assistance from all of the authors. D.Y.C., M.J.K, J.M.Y. and J.E.P. constructed the experimental setup for the fuel cell tests. Y.-H.C. and H.C. contributed to the planning and coordination of the project. D.Y.C. contributed to discussions of the results. Y.-H.C. and Y.-E.S. coordinated and supervised the overall project.

## Supplementary Material

Supplementary Information Supplementary Information

## Figures and Tables

**Figure 1:**
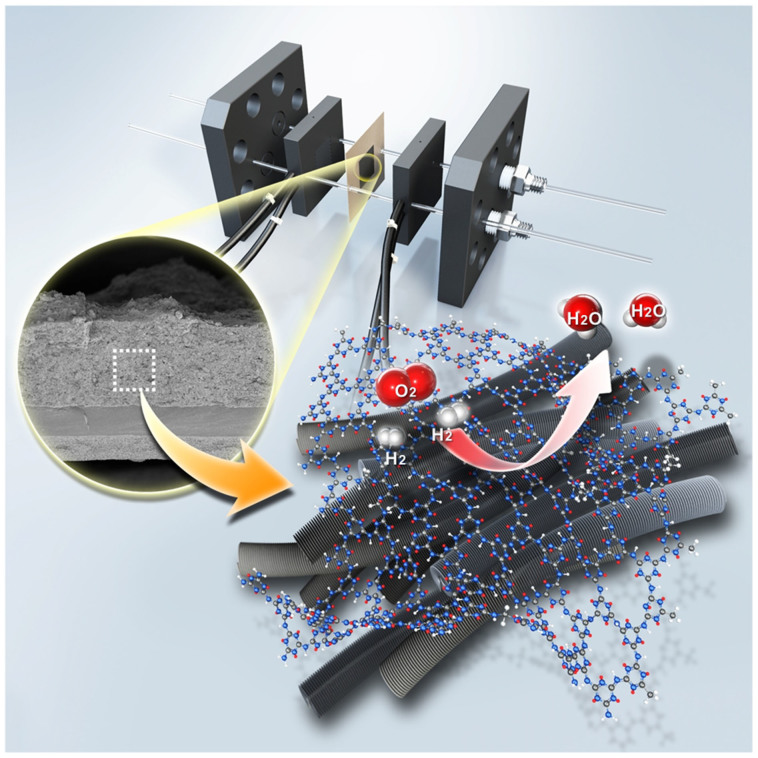
Conceptual diagrams of the g-CN-CNF-based MEA and the single cell. The inset shows a cross-section Fe-SEM image of the g-CN-based MEA.

**Table 1:** Performance characteristics of g-CN-CNF-700-based PEMFCs and AEMFCs

		Current density at 0.6 V (mA cm^−2^)	Max. power density (mW cm^−2^)
PEMFC	Commercial Pt/C	941 (100%)	890 (100%)
	g-CN-CNF-700	649 (69.0%)	464 (52.1%)
AEMFC	Commercial Pt/C	308 (100%)	222 (100%)
	g-CN-CNF-700	248 (80.5%)	171 (77.0%)
