# Supplementary material for: Facile and Gram-scale Synthesis of Metal-free Catalysts: Toward Realistic Applications for Fuel Cells
Source: Sci Rep. 2015 Mar 2;5:8376. doi: 10.1038/srep08376 (PMC4345340; doi:10.1038/srep08376)
Supplement: Supplementary Information [file srep08376-s1.pdf]

Supplementary Information

Facile and Gram-scale Synthesis of Metal-free Catalysts: Toward Realistic Applications for Fuel Cells

Ok-Hee Kim[1], Yong-Hun Cho[2], Dong Young Chung[3,4], Minjeong Kim[3,4], Ji Mun Yoo[3,4], Ji Eun Park[3,4], Heeman Choe[2] and Yung-Eun Sung[3,4]

[1] Department of Science, Republic of Korea Naval Academy, Jinhae-gu, Changwon-si, 645-797, South Korea

[2] School of Advanced Materials Engineering, Kookmin University, Seoul 136-702, South Korea

[3] Center for Nanoparticle Research, Institute for Basic Science (IBS), Seoul 151-742, South Korea

[4] School of Chemical and Biological Engineering, Seoul National University, Seoul 151-742, South Korea

Correspondence and requests for materials should be addressed to Y.-H. C. (E-mail: yhun00@kookmin.ac.kr) or to. Y.-E. S. (E-mail: ysung@snu.ac.kr)

Supplementary Note

Electrochemical characterization The electrochemical measurements were carried out using an Autolab general-purpose electrochemical system (Eco Chemie). Glassy carbon electrodes (0.196 cm^2) were coated with catalyst inks for use as the working electrode, and Pt wire and Ag/AgCl electrodes were used as the counter and reference electrodes, respectively. All of the potentials are quoted with respect to a reversible hydrogen electrode (RHE) through the hydrogen oxidation reaction. The ORR activities and methanol tolerances of the catalysts were measured using an RDE in 0.1 M KOH and 0.5 M CH_3OH saturated with oxygen gas at 293 K. LSV was performed from 1.0 V to 0.1 V vs. a reference hydrogen electrode at a scan rate of 5 mV s^{-1} and a rotation rate from 400 rpm to 1600 rpm. The ORR activities under acidic media were measured in a 0.1 M $HClO_4$ solution using the same method. The non-noble electrocatalyst was loaded at 98.4 μg (Pt loading was 28 μg, commercial E-TEK 20 wt%). The Koutecky-Levich plots were obtained by a linear fitting of the reciprocal rotating speed plotted as a function of the reciprocal current density collected at −0.6 V. The electron transfer numbers involved in the typical ORR process were calculated from the slope of the Koutecky-Levich equation:

$$B=0.2\ nFA v^{-1/6} C_{O2} D_{O2}^{2/3},$$

where n is the number of electrons transferred per oxygen molecule, F is the Faraday constant (96485 C mol^{-1}), D_{O2} is the diffusion coefficient of O_2 in 0.1 M KOH (1.9×10^{-5} cm s^{-1}), v is the kinetic viscosity, and C_{O2} is the concentration of O_2 (1.2×10^{-3} mol L^{-1}). The constant 0.2 is used when the rotating speed is expressed in units of rpm. The calculated electron transfer numbers are listed in the inset of Fig. S7b. The ADT was conducted according to the DOE protocol in the potential window from 0.6 V to 1.0 V (RHE) under Ar using a scan rate of 20 mV s^{-1} (Fig. S7).

Membrane electrode assembly (MEA) fabrication and single-cell test *1) (PEMFCs, Fig. 7a):* The typical MEA fabrication procedure has been described in detail elsewhere.[29] Briefly, a Nafion® 212 membrane (DuPont) was used as the ion-conducting membrane, which was immersed in a 2.5% H_2O_2 solution at 80 °C for 1 h and then rinsed in boiling deionized water for another 1 h for purification. The membrane was then boiled in a 0.5 M H_2SO_4 solution for 1 h and rinsed again in deionized water for protonation. The g-CN-CNF-700 and a 40 wt% Pt/C commercial catalyst (Johnson Matthey Co.) were used as the cathode and anode catalysts, respectively. The Pt/C catalyst was dispersed in a mixture of isopropyl alcohol, deionized water, and perfluorosulfonic acid ionomer (Aldrich, 5 wt% Nafion® ionomer) to prepare the catalyst ink. The ink was ultrasonicated for 10 min and sprayed onto both sides of a Nafion® 212 membrane. The volumes of the catalyst inks were carefully controlled so that they could be deposited with loadings of 0.2 mg cm^{-2} and 5.0 mg cm^{-2} for the anode and cathode, respectively. For comparison, a reference electrode

composed of 40 wt% Pt/C (Johnson Matthey Co.) was used as the anode catalyst with a loading of 0.2 mg cm^{-2} and as the cathode catalyst with a loading of 0.1 mg cm^{-2}. Carbon paper containing microporous layers (MPLs; 35BC, SGL) was used as a gas diffusion layer (GDL); it was placed on both the cathode and anode sides of the membrane. The MEAs were inserted into a single-cell unit containing a graphite plate with a serpentine gas flow channel (5 cm^2 area). A single-cell unit was assembled with eight screws tightened with a torque of 7.5 N·m. Activation and polarization tests of the assembled single cells (CNL-PEM005-01, CNL Energy) were carried out using the current sweep-hold method with a fuel cell test system (CNL Energy). The current density was swept at rate of 10 mA cm^{-2} s^{-1} and was maintained for 10 min after each of the following values was obtained: 0.5, 1.0, 1.5, 2.0, 2.5, 3.0, and 4.0 A cm^{-2}. During activation, the current was reset to zero when the cell voltage reached 0.30 V. Polarization curves (Fig. 7) were measured by the current-sweep method using the PEMFC test system. The test was conducted using fully humidified H_2/O_2, which were supplied to the anode and cathode, respectively, during the activation and polarization tests. The total outlet pressure was 150 kPa with an anode stoichiometry of 2 and a cathode stoichiometry of 9.5. The cell temperature was maintained at 80 °C during activation and at room temperature during the polarization test. *In situ* electrochemical impedance spectra of single cells were obtained under faradaic conditions after the polarization tests. The cell voltages used in the EIS measurements were 0.4, 0.6, and 0.8 V with a 10 mV amplitude; measurements were performed over the frequency range of 100 kHz–100 MHz using a potentiostat/galvanostat (IM-6, Zahner) (Fig. S8).

2) AEMFCs (Figure 7b): An anion exchange membrane was purchased from an anonymous company (details are withheld because of the company's material transfer agreement). The g-CN-CNF-700 and 40 wt% Pt/C (Johnson Matthey Co.) were used as the cathode and anode catalysts, respectively. The catalyst was dispersed in a mixture of isopropyl alcohol, deionized water, and anion exchange polymer (AS-4 ionomer, Tokuyama Co., Japan) to prepare the catalyst ink. The latter was stirred and ultrasonicated for 30 min and then sprayed onto the anion exchange membrane. The volume of anode catalyst ink was controlled such that it was deposited at 0.5 mg cm^{-2}, and the loadings of the cathode g-CN-CNF-700 and Pt/C catalysts were 2 mg cm^{-2} and 0.1 mg cm^{-2}, respectively. Carbon paper containing MPLs (35BC, SGL) was used as GDL, which was placed on both the cathode and anode sides of the membrane. The MEAs were inserted into the single-cell unit with a graphite plate containing a serpentine gas flow channel (5 cm^2 area). A single-cell unit was assembled with eight screws, using a tightening torque of 8 N·m. The polarization curves were measured by the current-sweep method with the fuel cell test system (CNL Energy). The test was conducted using fully humidified H_2/O_2, which were supplied to the anode and cathode, respectively. The total outlet pressure was 150 kPa with an anode stoichiometry of 2 and cathode stoichiometry of 9.5 for O_2. The cell temperature was maintained at 50 °C during the polarization tests..

Supplementary Figure

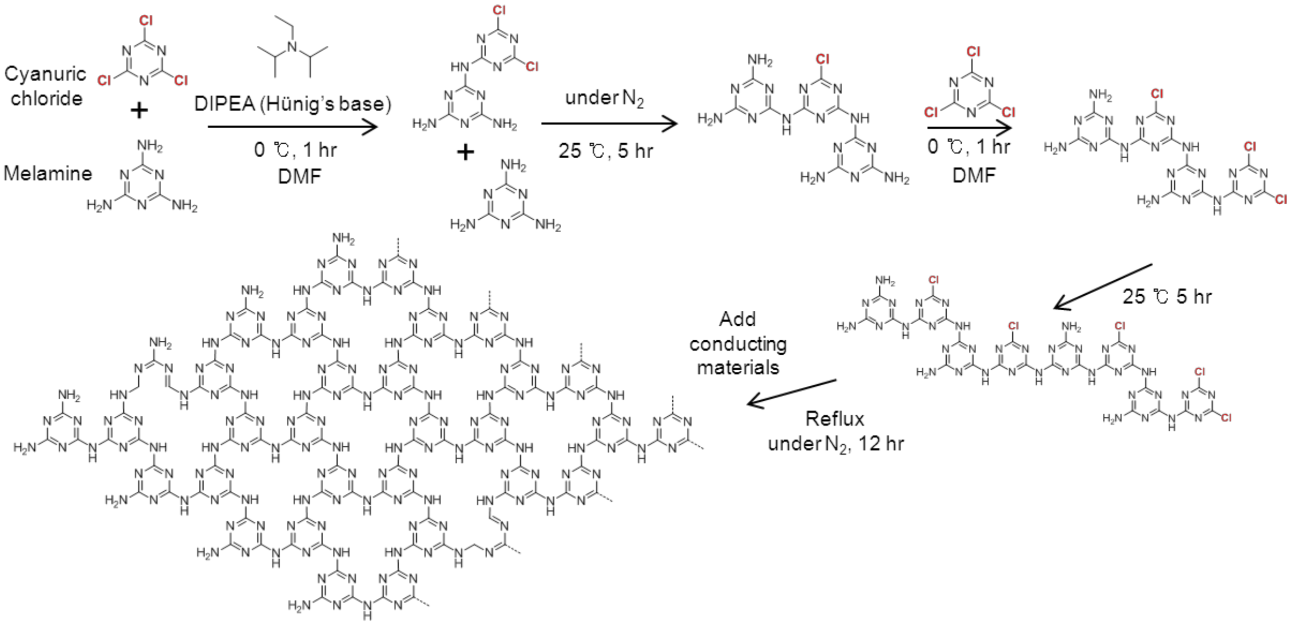

Supplementary Figure S1. Condensation reactions of melamine and cyanuric chloride to produce extended networks of g-CN.

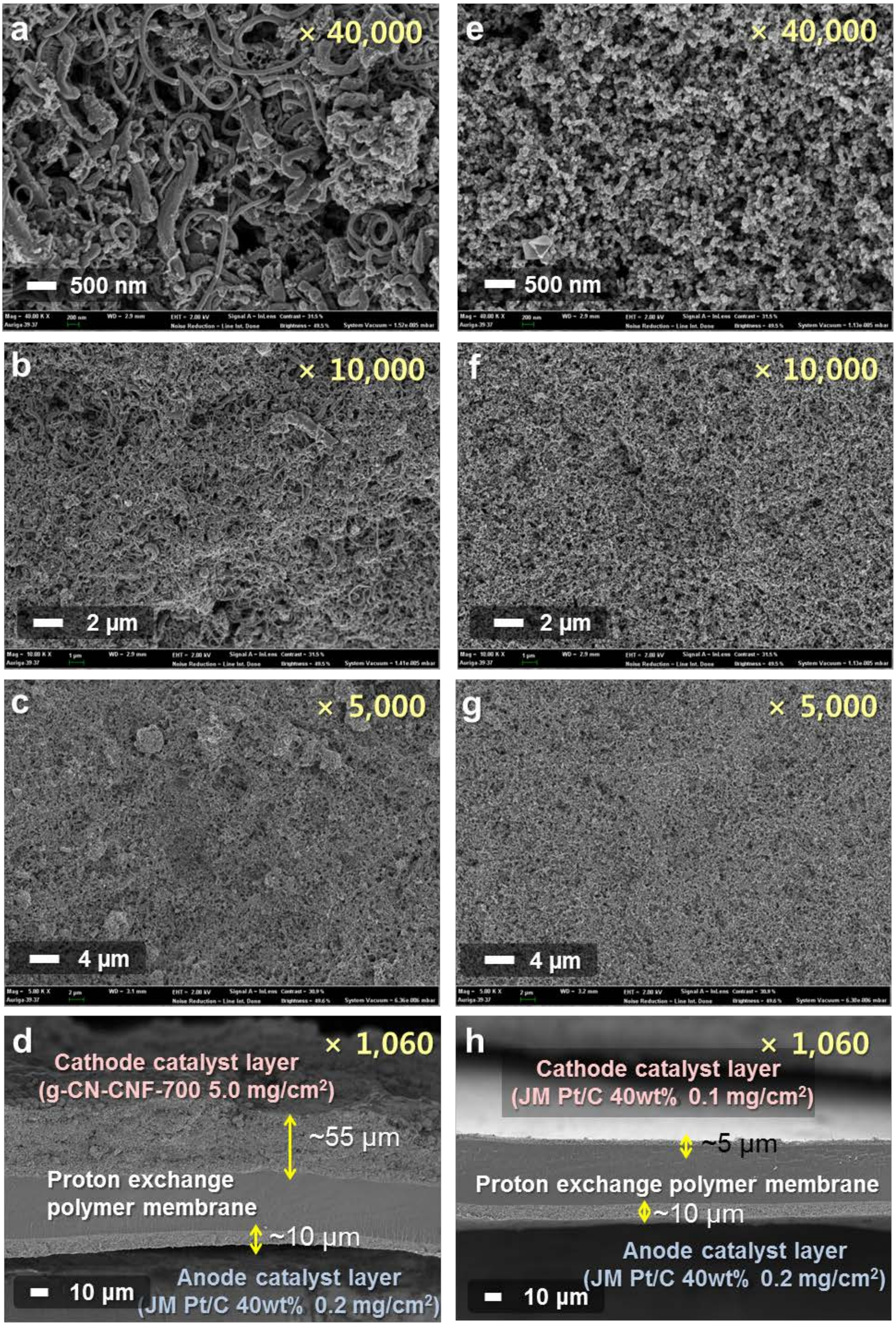

Supplementary Figure S2. FE-SEM images of g-CN-CNF and commercial Pt/C-based MEA in PEMFCs. a-c) Surfaces and d) cross-sectional images of g-CN-CNF-based MEA in PEMFCs; e-g) surfaces and d) cross-sectional images of commercial Pt/C-based MEA in PEMFCs..

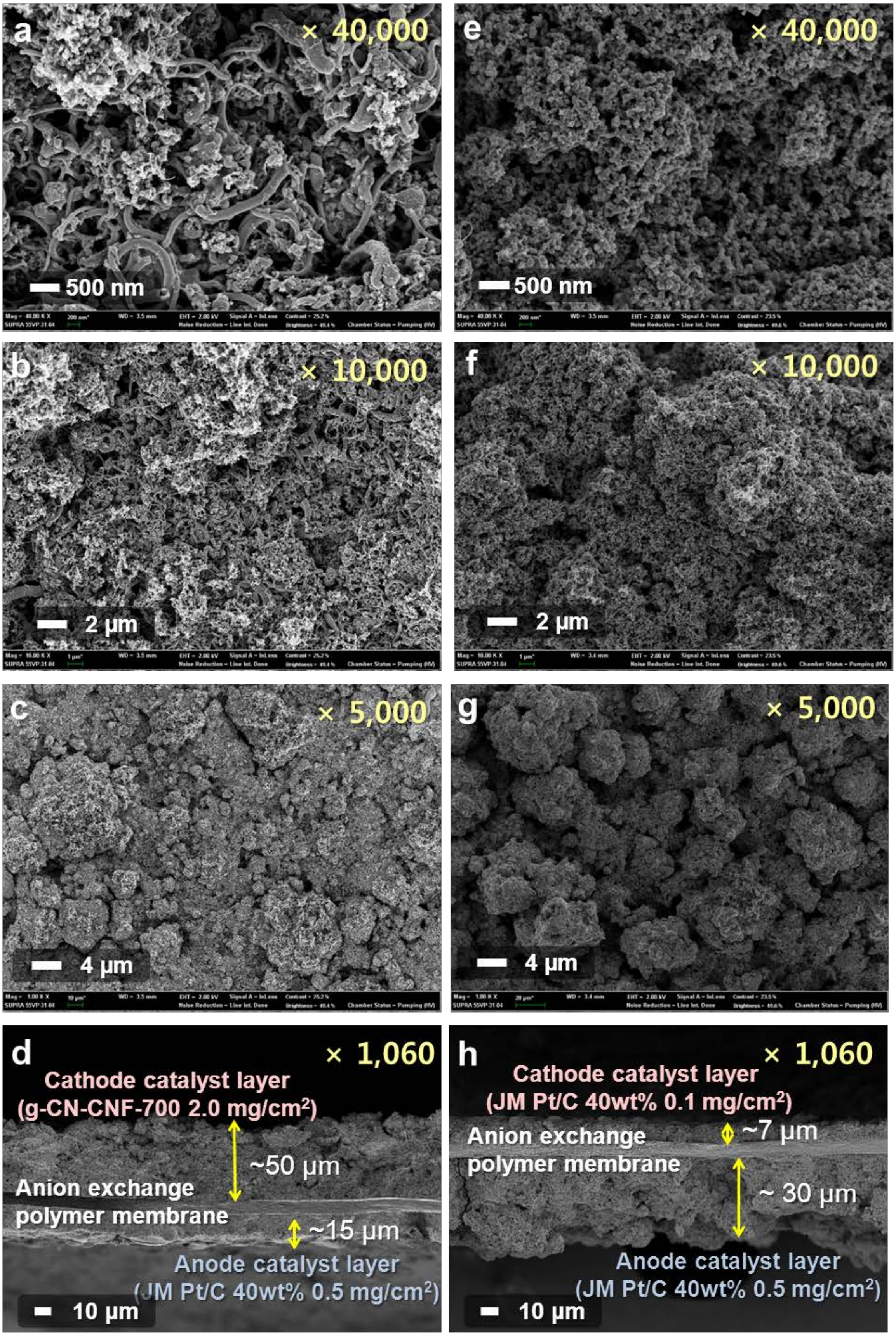

***Supplementary Figure S3*.** FE-SEM images of g-CN-CNF and commercial Pt/C-based MEA in AEMFCs. a-c) Surfaces and d) cross sectional images of g-CN-CNF-700-based MEA; e-g) surfaces and h) cross-sectional images of commercial Pt/C catalyst layers in AEMFCs.

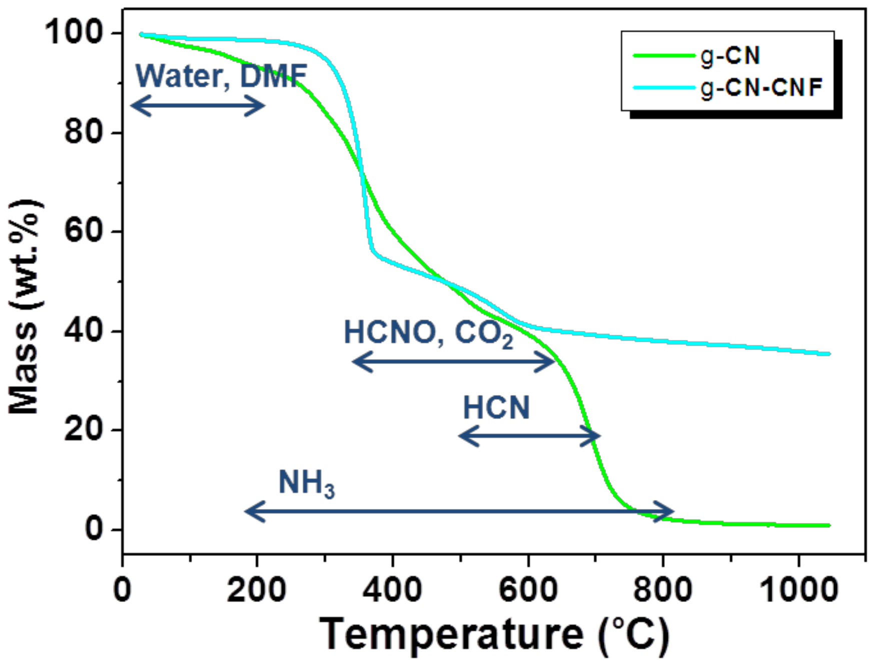

***Supplementary Figure S4*.** TGA weight loss curve of g-CN and g-CN-CNF measured in N_2 with rate of 5 °C/min.

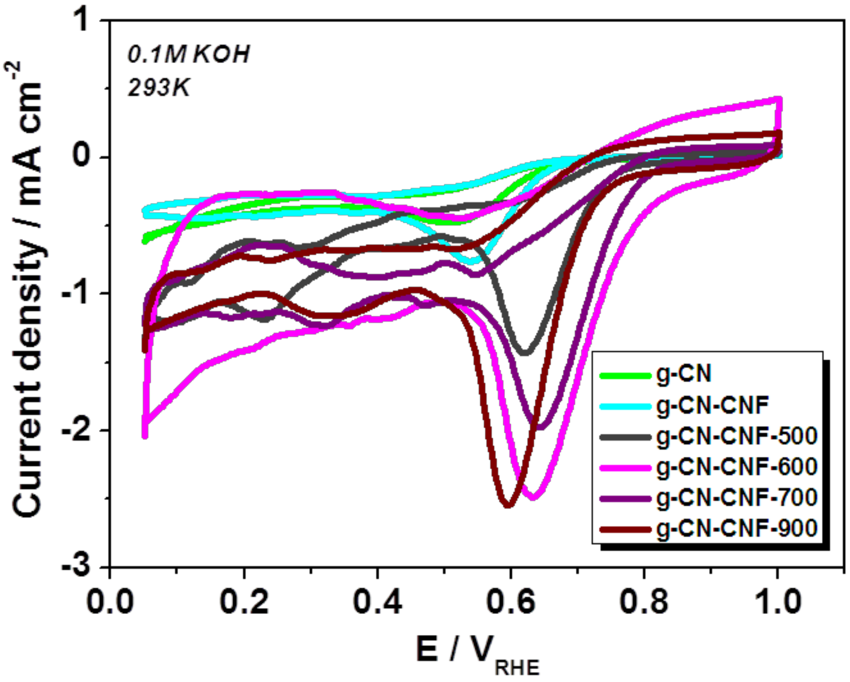

Supplementary Figure S5. Cyclic voltammetry performed for g-CN and g-CN-CNF-500/600/700/900 in O_2 and N_2 in a 0.1 M KOH aqueous solution at room temperature.

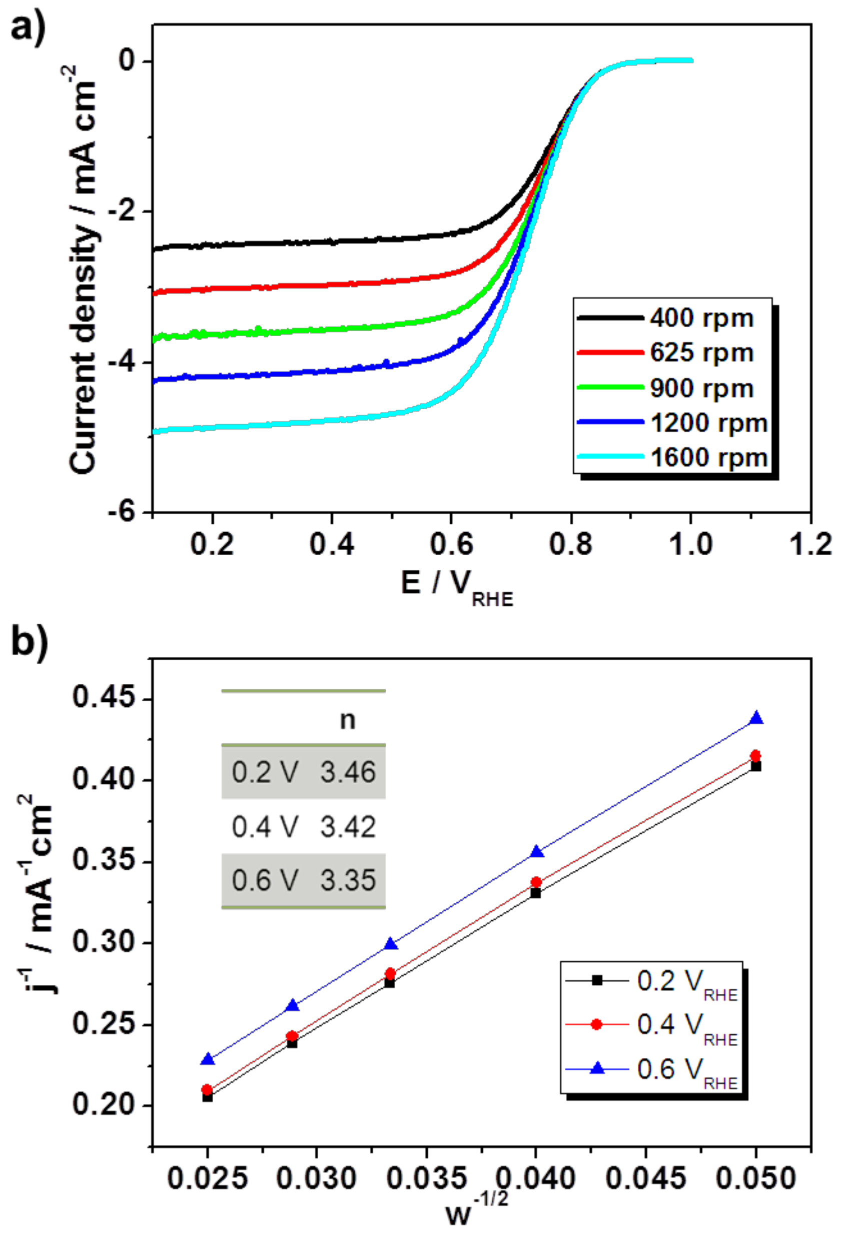

Supplementary Figure S6. . Koutecky–Levich plots of g-CN-CNF-700. a) LSVs obtained at various speeds of g-CN-CNF-700 from 400 rpm to 1600 rpm. b) The Koutecky-Levich plots of g-CN-CNF-700 were acquired at a current of 0.2, 0.4, and 0.6 V.

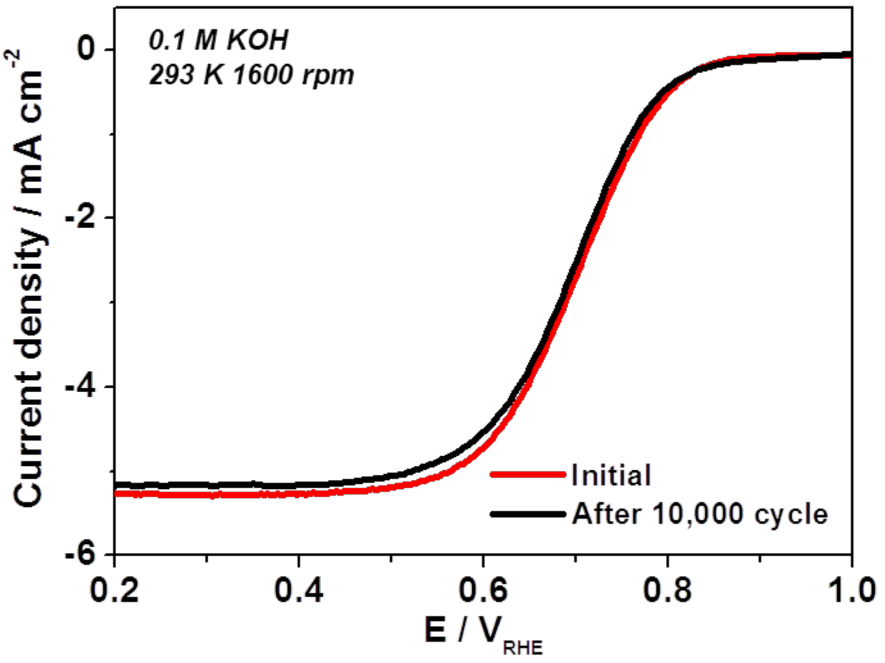

Supplementary Figure S7. Long-term stability test of g-CN-CNF-700. The accelerated durability test (ADT) was conducted based on the DOE protocol from 0.6V to 1.0 V (RHE) under Ar with a scan rate of 20 mV s^{-1}. After completion of initial LSV measurement and continuous potential cycling ~10,000 times; then the final LSV was measured again.

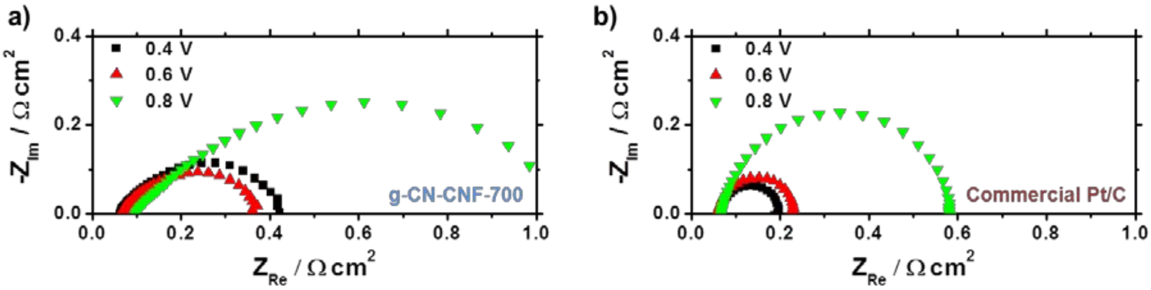

c)

Voltage	Ohmic resistance (R_{ohm}, mΩ cm^2)		Charge transfer resistance (R_{ct}, mΩ cm^2)	
	g-CN-CNF-700	Commercial Pt/C	g-CN-CNF-700	Commercial Pt/C
0.4 V	63.92	65.75	359.40	129.32
0.6 V	66.45	60.28	294.34	167.08
0.8 V	99.13	68.15	968.82	515.80

Supplementary Figure S8. *In-situ* EIS of (a) the g-CN-CNF-700-based MEA and b) commercial Pt/C-based MEA at 0.4 V (black square ■), 0.6 V (red triangle ▲), and 0.8 V (green triangle ▼) under operating conditions of 100% relative humidity at 80 °C in PEMFCs, c) the ohmic and charge transfer resistances of the cell using a g-CN-CNF-700 electrode and commercial Pt/C MEA from EIS.

Supplementary Table S1. Nitrogen properties, binding energy, atomic ratio and N content of g-CN materials.

Material		Property			N content [b] (wt%)
		N1	N2	N3	
g-CN-CNF	Binding energy (eV)	398.80	400.34	-	26.34
	Ratio (%) [a]	66.7	33.2	-	
g-CN-CNF-500	Binding energy (eV)	399.00	400.20	401.19	10.31
	Ratio (%)	59.8	30.9	9.4	
g-CN-CNF-700	Binding energy (eV)	398.93	400.44	401.36	1.91
	Ratio (%)	69.3	10.3	20.4	
g-CN-CNF-900	Binding energy (eV)	398.54	400.34	401.17	0.81
	Ratio (%)	61.6	15.2	23.3	

[a]Calculated using Avantage software.
[b]From EA (Elemental Analyzer) result.
